# Supplementary material for: Global hypo-methylation in a proportion of glioblastoma enriched for an astrocytic signature is associated with increased invasion and altered immune landscape
Source: eLife. 2022 Nov 22;11:e77335. doi: 10.7554/eLife.77335 (PMC9681209; doi:10.7554/eLife.77335)
Supplement: Figure 2—source data 1. [file elife-77335-fig2-data1.zip › Figure_2_source_data_1/Figure_2B/homerResults/motif2.similar.html]

motif2

## Information for motif2

A
C
G
T
A
T
C
G
A
C
G
T
A
C
T
G
A
C
T
G
A
C
T
G
A
T
G
C
C
G
T
A
  
Reverse Opposite:  

A
C
G
T
A
T
C
G
G
T
A
C
A
G
T
C
T
G
A
C
C
G
T
A
A
T
G
C
G
T
C
A
  

|  |  |
| --- | --- |
| p-value: | 1e-88 |
| log p-value: | -2.039e+02 |
| Information Content per bp: | 1.890 |
| Number of Target Sequences with motif | 107.0 |
| Percentage of Target Sequences with motif | 4.63% |
| Number of Background Sequences with motif | 1.9 |
| Percentage of Background Sequences with motif | 0.54% |
| Average Position of motif in Targets | 92.2 +/- 56.8bp |
| Average Position of motif in Background | 50.4 +/- 27.6bp |
| Strand Bias (log2 ratio + to - strand density) | 0.1 |
| Multiplicity (# of sites on avg that occur together) | 1.08 |
| Motif File: | file (matrix) reverse opposite |

### Similar de novo motifs found

|  |  |  |  |  |  |  |  |
| --- | --- | --- | --- | --- | --- | --- | --- |
| Rank | Match Score | Redundant Motif | P-value | log P-value | % of Targets | % of Background | Motif file |
| 1 | 0.641 | A T G C A G C T A T G C G A C T A T C G A C G T C A T G C A T G C T A G A T C G | 1e-69 | -159.699928 | 3.94% | 0.32% | motif file (matrix) |
| 2 | 0.631 | A G T C A G T C A C G T A G T C A G T C A G T C A G T C C G T A A T C G C T G A A C T G A G T C | 1e-55 | -126.906696 | 6.75% | 1.65% | motif file (matrix) |
| 3 | 0.634 | A G T C G C T A G A T C A C G T A G T C A G T C A G T C A G T C C G T A A G T C C G A T A T G C A G T C A G T C G C T A | 1e-48 | -110.873277 | 4.11% | 0.60% | motif file (matrix) |
| 4 | 0.683 | A G T C A T G C A C T G C G T A A G T C C G T A | 1e-44 | -102.259882 | 4.72% | 0.98% | motif file (matrix) |
| 5 | 0.653 | A G T C A C G T G T C A A C G T T C A G A T C G A C T G A G T C | 1e-33 | -78.123304 | 3.38% | 0.85% | motif file (matrix) |
| 6 | 0.600 | A C T G A G C T A T C G A C G T A G C T A C T G | 1e-29 | -66.947554 | 18.35% | 10.63% | motif file (matrix) |
